# Adherence to Brain Trauma Foundation guidelines for management of traumatic brain injury patients: study protocol for a systematic review and meta-analysis

**DOI:** 10.1186/s13643-015-0140-1

**Published:** 2015-11-05

**Authors:** Yahya H Khormi, Ibrahim Gosadi, Sandra Campbell, Ambikaipakan Senthilselvan, Cian O’kelly, David Zygun

**Affiliations:** Division of Neurosurgery, Department of Surgery, University of Alberta and the University of Alberta Hospital, Edmonton, AB Canada; Faculty of medicine, Jazan University, Jazan, Saudi Arabia; Prince Sattam Chair for Epidemiology and Public Health Research, Department of Family and Community Medicine, College of Medicine, King Saud University, 11541, BO 2454, Riyadh, Kingdom of Saudi Arabia; John W. Scott Health Sciences Library, 2 K3.26 Walter C. Mackenzie Health Sciences Centre, University of Alberta, Edmonton, AB T6H 5L8 Canada; School of Public Health, 3-276 Edmonton Clinic Health Academy, University of Alberta, Edmonton, AB Canada; Division of Neurosurgery, Department of Surgery, University of Alberta, Edmonton, AB Canada; Division of Critical Care Medicine, 2-124 Clinical Sciences Building, University of Alberta, Edmonton, AB Canada

**Keywords:** Traumatic brain injuries, TBI, Brain injury guidelines, Brain Trauma Foundation guidelines

## Abstract

**Background:**

Traumatic brain injury (TBI) is a leading cause of death and disability around the world. Management based on Brain Trauma Foundation (BTF) guidelines is widely accepted and thought to improve outcome. The objectives of this systematic review are to give an overview of adherence to the BTF guidelines, describe factors influencing adherence, and study the effect of guideline-based management on outcome.

**Methods/design:**

We will search electronic bibliographic databases: PROSPERO, Medline, EMBASE, SCOPUS, NHS, CINAHL, Cochrane Database, and ProQuest Dissertations and Theses Global. Two investigators will independently screen all titles, abstracts, and articles and select Randomized Controlled Trial (RCT), cohort studies, case-control studies, and case series reporting the adherence rate, factors influencing adherence, and mortality or morbidity. These investigators will also independently extract data using a pre-designed and pilot-tested standardized electronic data extraction form and assess the risk of bias. We will exclude pediatric and military-related TBI studies, studies that included fewer than ten patients or addressed adherence to pre-hospital guidelines. Narrative synthesis and if appropriate, quantitative meta-analysis clustered by type of recommendation will be reported.

**Discussion:**

This study is expected to demonstrate the current level of professionals’ adherence to BTF guidelines in patients with severe traumatic brain injury, it will describe the factors influencing adherence, which may provide valuable input for development of strategies to successfully increase adherence. In addition, if the studies are sufficiently homogenous, it will describe the effect of these guidelines on patient outcome.

**Systematic review registration:**

PROSPERO CRD42015017794

**Electronic supplementary material:**

The online version of this article (doi:10.1186/s13643-015-0140-1) contains supplementary material, which is available to authorized users.

## Background

Traumatic brain injury (TBI) is a leading cause of death and disability around the world [1, 2]. In the USA, the prevalence of TBI is estimated to be 2 % in the general population [3]. The mortality rate was reported to be 18.4 per 100,000 persons with annual average of 53,014 deaths [4]. A report from the Canadian Institute for Health Information (CIHI) report indicated that there were 16,811 hospitalizations annually for TBI with 1368 (8 %) related deaths [5]. Among residents in a large Canadian health region, the annual incidence of severe TBI was 11.4 per 100,000 persons with a mortality rate of 5.1 per 100,000 persons per year [6].

Clinical practice guidelines are developed to improve quality of care decrease discrepancy in practice and ensure that evidence is followed [7]. Mostly, these guidelines are developed and distributed by well-recognized organizations. A guideline consists of systematically developed recommendations to guide practitioners in choosing the appropriate health care decision for specific clinical circumstances [8]. A guideline recommendation is defined as “any statement that promotes or advocates a particular course of action in clinical care” [9]. In the treatment of TBI, guidelines are proposed to be an important aspect of patient management.

There are several published guidelines in the management of TBI from different countries. These guidelines target different aspects of TBI management including management during pre-hospital at the emergency department, in-hospital and intensive care unit, indications for surgical management, and computed tomography (CAT) scan of the head [10–14].

Internationally, Brain Trauma Foundation (BTF) guidelines are widely disseminated. They have been translated into over 15 different languages and applied in Europe, South America, and some parts of China [12]. The BTF maintains and revises several TBI guidelines on an approximate 5-year cycles, including *Guidelines for Prehospital Management of Traumatic Brain Injury*, *Guidelines for the Management of Severe Traumatic Brain Injury*, *Guidelines for the Surgical Management of Traumatic Brain Injury*, *Guidelines for the Acute Medical Management of Severe Traumatic Brain Injury in Infants, Children, and Adolescents*, and *Guidelines for the Field Management of Combat Related Head Trauma and Early Indicators of Prognosis of Severe Traumatic Brain Injury*. These guidelines are developed and maintained through a collaborative agreement with the American Association of Neurological Surgeons (AANS) and the Congress of Neurological Surgeons (CNS), and in collaboration with the AANS/CNS Joint Section on Neurotrauma and Critical Care, European Brain Injury Consortium, and other stakeholders in TBI patient outcome [12].

*Guidelines for Management of Severe Traumatic Brain Injury* addresses key topics useful for in-hospital medical management of severe TBI in adult patients with a Glasgow Coma Scale (GCS) score of 3–8. These include blood pressure and oxygenation hyperosmolar therapy, prophylactic hypothermia, infection prophylaxis, deep vein thrombosis prophylaxis, intracranial pressure monitoring, cerebral perfusion thresholds, brain oxygen monitoring and thresholds, anesthetics, analgesics and sedatives, nutrition, antiseizure prophylaxis, and hyperventilation through steroids use. In 2007, the third edition of these *Guidelines* was released following the first and second editions in 1995 and 2000. [12, 15, 16].

*Guidelines for the Surgical Management of Traumatic Brain Injury* addresses acute surgical management of TBI including acute epidural and subdural hematomas, parenchymal mass lesions, depressed skull fractures through posterior fossa lesions with focus on indications, technique, and timing of surgery. These *Guidelines* were published in 2006 [13].

Studies suggest that implementation and strict adherence to BTF guidelines results in improvement in the neurological outcomes and reduction in mortality from severe traumatic brain injury [17, 18]. However, there is still significant variability and inconsistency in the management of traumatic brain injury patients [19, 20]. This review will be the first systematic review assessing the adherence to BTF guidelines and its effect on outcome.

### Objectives

The first objective of this study is to present a systematic review of adherence by practitioners to the BTF guidelines for the management of severe TBI. The second objective is to explore the factors influencing adherence to the guidelines. Identification of these factors may provide valuable insight into the development of strategies to increase the adherence. The third objective is to study the outcome of guideline-based management in comparison to non-guideline based management to determine the effectiveness of these guidelines.

## Methods/design

### Protocol and study overview

Methods of this systematic review and meta-analysis have been developed in accordance with the Preferred Reporting Items for Systematic Reviews and Meta-Analyses (PRISMA) [21] and the Meta-Analysis of Observational Studies in Epidemiology (MOOSE) guidelines [22]. We will begin by developing a comprehensive database containing all published literature that addresses adherence to BTF guidelines in the management of severe TBI. This protocol has been registered in the PROSPERO International Prospective Register of Systematic Reviews (ID: CRD42015017794).

### Selection criteria

#### Population

The population of interest will include adult (≥18 years old) hospitalized patients with blunt TBI. Whenever outcome measures are available, the patients who were treated based on the BTF guideline will be compared to the patients who were not treated based on the BTF guideline. Additionally, the population of this study will include the practitioners, mainly the neurosurgeons and critical care physicians, who will be assessed for adherences to guidelines. The assessed guidelines will be (a) in-hospital guidelines regarding blood pressure and oxygenation, hyperosmolar therapy, prophylactic hypothermia, infection prophylaxis, deep vein thrombosis prophylaxis, indications for intracranial pressure monitoring, intracranial pressure monitoring technology, intracranial pressure thresholds, cerebral perfusion thresholds, brain oxygen monitoring and thresholds, anesthetics, analgesics, sedatives, nutrition, antiseizure prophylaxis, hyperventilation, and steroids. (b) Guidelines for surgical management for acute epidural and subdural hematomas, parenchymal lesions, posterior fossa mass lesions, and depress cranial fractures. We will exclude (1) studies addressed adherence to pre-hospital guidelines (the result from studies on pre-hospital management may not reflect the adherence because failure to achieve target recommendation may be an indicator of severe injury), (2) studies focused on military/combat-related TBI, because the results would not be generalizable to the source population of civilian patients with TBI, and (3) studies with majority of pediatric patients.

#### Outcome

The main outcome will be the adherence rate with BTF guidelines. In addition, we will identify factors influencing the adherence to the BTF guidelines. The effectiveness of adherence with the BTF guidelines on several clinical outcomes will be assessed. The measured clinical outcomes will include mortality (ICU, in-hospital mortality) and morbidity (Glasgow Outcome Scale (GOS), Modified Rankin Scale (MRS), ventilation days, ICU stay, and hospital stay).

#### Study design

Original searches will include RCT cohort, case-control, and case series. We will exclude studies that included fewer than ten patients.

#### Search strategy

The primary search strategy was developed by the primary investigator (YK) and in collaboration with an expert searcher/librarian (SC). We will search the following electronic bibliographic databases: PROSPERO Medline (OVID), EMBASE (OVID), EBM Reviews—Cochrane Database of Systematic Reviews, EBM Reviews—ACP Journal Club, EBM Reviews—Database of Abstracts of Reviews of Effects, EBM Reviews—Cochrane Central Register of Controlled Trials, EBM Reviews—Cochrane Methodology Register, EBM Reviews—Health Technology Assessment, EBM Reviews—NHS Economic Evaluation Database, CINAHL Plus with Full Text, ProQuest Dissertations and Theses Full-text, SCOPUS, and Google Scholar using both controlled vocabulary (e.g., EMTREE and MeSH) and keywords to retrieve concepts including Brain Trauma Foundation or brain injur* and guideline* and adhere*. Searches will be limited to adult patients in non-military settings. Animal studies will be excluded. This systematic review will include searching gray literature, reviewing references lists, and contacting experts in the field. (See appendix in Additional file 1 for the final proposed MEDLINE, EMBASE, and EBM Reviews—Cochrane Database of Systematic Reviews search strategy).

#### Study selection

Two investigators (YK and IG) will independently screen all title abstracts and articles to identify study meeting inclusion/exclusion criteria. Inclusion disagreement will be discussed and resolved by consensus or arbitration by other researchers (CO and DZ).

### Data extraction

Two investigators (YK and IG) will independently extract data from eligible studies using a pre-designed and pilot-tested standardized electronic data extraction form. We will extract data on (1) publication details (year and language of publication name of the publishing journal and country in which the study was conducted). (2) Design: type of study (RCT, cohort, case-control, case series), study temporality (prospective, retrospective). (3) Study participant details: patient characteristics (age, sex, GCS, Injury severity score). (4) Data for percentage adherence to BTF guidelines. From each article, adherence percentages for each recommendation will be extracted. In case of a pre- and post-intervention design for evaluation of intervention (for example introducing a protocol or teaching program), only the post-intervention percentages will be extracted because our interest is in the current clinical practice. (5) Demographic and injury-related characteristics, which may influence adherence to the BTF guidelines: increase age, elevated blood alcohol level, normal CT scan, and planned neurosurgical intervention or other factors reported in the study will be extracted when a statistically significant relationship between these factors and adherence is demonstrated. (6) Outcomes including mortality or morbidity if they compared between patients treated according to the BTF guidelines and patients treated differently and 95% confidence interval are reported. Discrepancies will be discussed and resolved by consensus or arbitration by other researchers (CO and DZ).

### Quality assessment

#### Randomized Controlled Trials (RCT)

The quality will be assessed using the Cochrane Handbook “Risk of Bias” assessment tool [23]. Additionally, we will assess the quality of reporting using a checklist, which will be based on the CONSORT (Consolidated Standards of Reporting Trials).

#### Observational studies

The quality will be assessed using the Cochrane Risk Of Bias Assessment Tool: for Non- Randomized Studies of Interventions (ACROBAT-NRSI) [24] which evaluates the observational studies based on three domains: (1) Pre-intervention; evaluation of bias due to confounding and bias in selection of participants into the study. (2) At intervention: evaluation of bias in measurement of interventions. (3) Post-intervention: evaluation of biases due to departures from intended interventions bias due to missing data, bias in measurement of outcomes and bias in selection of the reported results. We will assess the quality of reporting of observational studies using a checklist, which will be based on the STROBE (Strengthening the Reporting of Observational Studies in Epidemiology) statement.

Two researchers (YK and IG) will address quality assessment of the included studies independently. Differences of opinion will be resolved by a discussion with other researchers (CO and DZ).

### Data synthesis

Narrative synthesis and where appropriate, quantitative meta-analysis will be used. Synthesis will be based on clustering the selected studies based on type of recommendation. Adherence to BTF-based protocol will be extracted as a separate category if the full description of the protocol and protocol adherence rate was reported. Data synthesis will include description of included studies.

The median adherence and interquartile range for each recommendation will be calculated as well as for overall adherence. Additionally, factors influencing adherence will be examined based on the type of recommendation.

### Calculation of pooled estimates of mortality among TBI patients managed based on BTF guidelines and patients managed differently

In preliminary search, odds ratio was used as measure of association in several studies, and we will also use the odds ratio as the summary measure of association in our study. If only the relative risk is reported in a selected study, we will transform the relative risk into an odds ratio using the method described by Deeks and Altman [25]. The cohort studies and RCTs will be pooled separately. We will conduct stratified analyses of pooled estimate of mortality by type of recommendation and outcome (for example in-hospital mortality intensive care unit mortality, 30 days mortality or 6 months mortality). We will examine heterogeneity separately in the pooled estimates by study design (RCT, observational) using the Cochran *Q* and *I*^2^ statistics [24]. In the presence of heterogeneity, random effects models will be used instead of fixed effects models to account for the expected variability beyond the chance and obtain pooled effect estimates across studies [26]. The pooled estimates obtained from these calculations will then be compared to determine if the results are different between experimental and cohort study designs. If the adjustment for confounding variables varies between studies, analysis will be stratified into two parts, one for studies adjusting for several confounding variables (e.g., age, GCS, injury severity score, pupillary response, and CAT scan head finding) and the other one for studies adjusting for a few confounding variables.

If an adequate number of studies are chosen for the meta-analysis, we will conduct meta-regression considering the following covariates: year of publication, country of origin, and study period.

Publication bias will also be assessed using funnel plot and the methods described by Begg and Egger [27, 28]. Meta-analysis will be performed using Review Manager software (RevMan5.3.5 Cochrane Collaboration) and regression analysis will be conducted using Stata Statistical Software version 13.1. (StataCorp LP, College Station, TX, USA).

All data will be extracted by two independent investigators (YK IG). To assess inter-rater reliability, the percent agreement will be calculated on adherence percentage for number of guideline recommendations by third investigator (AS).

## Discussion

This systematic review and meta-analysis will be the first systematic review summarizing relevant literature on guidelines for management of severe traumatic brain injury. In this review, we will demonstrate the current level of professionals’ adherence to BTF guidelines in patients with severe traumatic brain injury. In addition, we will describe the factors influencing adherence, which may provide valuable input for development of strategies to successfully increase adherence. Finally, we will describe the effect of these guidelines on patient outcome if data is sufficiently homogenous. Results of this review are expected to be available near the end of 2015.

The major strength of this systematic review will be the use of several electronic databases and other relevant sources based on established guideline for systematic review. An additional strength of the review will be the use of inter-rater reliability, standard protocol for reporting systematic reviews as well as quality assessment of the included studies. However, there are some limitations in this review. We may not be able to find the non-observational studies due to the nature of the measured effect. Furthermore, this review will be examining adherences with different recommendations at several locations using different clinical determinants. Therefore, a high level of heterogeneity will be expected and may limit our ability to perform meta-analysis.

## Additional file

Additional file 1:
**Appendix 1.** Proposed Medline search strategy; **Appendix 2.** Proposed Embase search strategy; **Appendix 3.** Proposed EBM Reviews—Cochrane Database of Systematic Reviews search strategy. (DOCX 24 kb)

## Abbreviations

BTF: Brain Trauma Foundation
TBI: traumatic brain injury
CINAHL: Cumulative Index to Nursing and Allied Health Literature
CENTRAL: Cochrane Central Register of Controlled Trials
RCT: Randomized Controlled Trial
AANS: American Association of Neurological Surgeons
CNS: Congress of Neurological Surgeons
GCS: Glasgow Coma Scale
MOOSE: Meta-analysis of Observational Studies in Epidemiology
PRISMA: Preferred Reporting Items for Systematic Reviews and Meta-Analyses
EBM: evidence-based medicine
ACP: American College of Physicians
NOS: Newcastle-Ottawa Scale
STROBE: Strengthening the Reporting of Observational studies in Epidemiology
