# Supplementary material for: Adherence to Brain Trauma Foundation guidelines for management of traumatic brain injury patients: study protocol for a systematic review and meta-analysis
Source: Syst Rev. 2015 Nov 5;4:149. doi: 10.1186/s13643-015-0140-1 (PMC4634601; doi:10.1186/s13643-015-0140-1)
Supplement: Additional file 1: — Appendix 1. Proposed Medline search strategy; Appendix 2. Proposed Embase search strategy; Appendix 3. Proposed EBM Reviews—Cochrane Database of Systematic Reviews search strategy. (DOCX 24 kb) [file 13643_2015_140_MOESM1_ESM.docx]

Additional file 1

Appendix 1

Proposed Medline search strategy

1     Brain Trauma Foundation.mp.
2     Guidelines for the Management of Severe Traumatic Brain Injury.mp.
3     Guidelines for the Surgical Management of Traumatic Brain Injury.mp.
4     Guidelines for Prehosptial Management of Traumatic Brain Injury.mp. [mp=title, abstract, original title, name of substance word, subject heading word, keyword heading word, protocol supplementary concept word, rare disease supplementary concept word, unique identifier]
5     (Prehosptial Management of Traumatic Brain Injury and guideline*).mp. [mp=title, abstract, original title, name of substance word, subject heading word, keyword heading word, protocol supplementary concept word, rare disease supplementary concept word, unique identifier]
6     Prehosptial Management of Traumatic Brain Injury.mp. [mp=title, abstract, original title, name of substance word, subject heading word, keyword heading word, protocol supplementary concept word, rare disease supplementary concept word, unique identifier]
7     Guideline* for the Surgical Management of Traumatic Brain Injury.mp.
8     (guideline* or protocol*).mp. and (complian* or comply* or adhere*).ti,ab. [mp=title, abstract, original title, name of substance word, subject heading word, keyword heading word, protocol supplementary concept word, rare disease supplementary concept word, unique identifier] 9     ((craniocerebral or head or cranium or skull or skulls or cerebrocranial or cranial) adj1 (injur* or trauma* or wound*)).ti,ab.
10     8 and 9
11     exp Guideline Adherence/ and 9
12     1 or 2 or 3 or 4 or 5 or 6 or 7 or 10 or 11
13     exp child/ or exp congenital/ or exp infant/ or exp adolescence/ or exp infant, newborn/ or exp child, preschool/ or (pediatric* or paediatric* or child* or newborn* or congenital* or infan* or baby or babies or neonat* or pre-term or premature birth or NICU or preschool* or pre-school* or kindergarten* or elementary school$ or nursery school$ or schoolchild* or toddler$ or boy or boys or girl* or middle school* pubescen* or juvenile* or teen* or youth* or high school* or adolesc* or pre-pubesc*).mp. or (child* or adolesc* or pediat* or paediat*).jn.
14     12 and 13
15     12 not 14
16     limit 14 to "all adult (19 plus years)"
17     15 or 16
18     remove duplicates from 17

Appendix 2

Proposed Embase search strategy

1     Brain Trauma Foundation.mp.
2     Guidelines for the Management of Severe Traumatic Brain Injury.mp.
3     Guidelines for the Surgical Management of Traumatic Brain Injury.mp.
4     Guidelines for Prehosptial Management of Traumatic Brain Injury.mp. [mp=title, abstract, subject headings, heading word, drug trade name, original title, device manufacturer, drug manufacturer, device trade name, keyword]
5     (Prehosptial Management of Traumatic Brain Injury and guideline*).mp. [mp=title, abstract, subject headings, heading word, drug trade name, original title, device manufacturer, drug manufacturer, device trade name, keyword]
6     Prehosptial Management of Traumatic Brain Injury.mp. [mp=title, abstract, subject headings, heading word, drug trade name, original title, device manufacturer, drug manufacturer, device trade name, keyword]
7     Guideline* for the Surgical Management of Traumatic Brain Injury.mp.
8     ((guideline* or protocol*) and (complian* or comply* or adhere*)).ti,ab.
9     exp *practice guideline/
10   exp physician/
11     exp *protocol compliance/
12     9 or 11
13     10 and 12
14     8 or 13
15     brain injury/ or acquired brain injury/ or brain concussion/ or brain stem injury/ or cerebellum injury/ or traumatic brain injury/
16     head injury/
17     ((craniocerebral or head or cranium or skull or skulls cerebrocranial or cranial) adj1 (injur* or trauma* or wound*)).mp. [mp=title, abstract, subject headings, heading word, drug trade name, original title, device manufacturer, drug manufacturer, device trade name, keyword]
18     15 or 16 or 17
19     14 and 18
20     1 or 2 or 3 or 4 or 5 or 6 or 7 or 19
21     exp child/ or exp "congenital, hereditary, and neonatal diseases and abnormalities"/ or exp infant/ or exp adolescence/ or exp infant, newborn/ or exp child, preschool/ or (pediatric* or paediatric* or child* or newborn* or congenital* or infan* or baby or babies or neonat* or pre-term or premature birth or NICU or preschool* or pre-school* or kindergarten* or elementary school* or nursery school* or schoolchild* or toddler* or boy or boys or girl* or middle school* or pubescen* or juvenile* or teen* or youth* or high school* or adolesc* or pre-pubesc*).mp. or (child* or adolesc* or pediat* or paediat*).jn.
22     20 not 21
23     20 and 21
24     limit 23 to (adult <18 to 64 years> or aged <65+ years>)
25     22 or 24
26     remove duplicates from 25

Appendix 3

Proposed EBM Reviews - Cochrane Database of Systematic Reviews search strategy

1     Brain Trauma Foundation.mp.
2     Guidelines for the Management of Severe Traumatic Brain Injury.mp.
3     Guidelines for the Surgical Management of Traumatic Brain Injury.mp.
4     Guidelines for Prehosptial Management of Traumatic Brain Injury.mp. [mp=ti, ab, tx, kw, ct, ot, sh, hw]
5     (Prehosptial Management of Traumatic Brain Injury and guideline*).mp. [mp=ti, ab, tx, kw, ct, ot, sh, hw]
6     Prehosptial Management of Traumatic Brain Injury.mp. [mp=ti, ab, tx, kw, ct, ot, sh, hw]
7     Guideline* for the Surgical Management of Traumatic Brain Injury.mp.
8     (guideline* or protocol*).mp. and (complian* or comply* or adhere*).ti,ab. [mp=ti, ab, tx, kw, ct, ot, sh, hw]
9     ((craniocerebral or head or cranium or skull or skulls or cerebrocranial or cranial) adj1 (injur* or trauma* or wound*)).ti,ab.
10     8 and 9
11     exp Guideline Adherence/ and 9
12     1 or 2 or 3 or 4 or 5 or 6 or 7 or 10 or 11
13     exp child/ or exp congenital/ or exp infant/ or exp adolescence/ or exp infant, newborn/ or exp child, preschool/ or (pediatric* or paediatric* or child* or newborn* or congenital* or infan* or baby or babies or neonat* or pre-term or premature birth or NICU or preschool* or pre-school* or kindergarten* or elementary school$ or nursery school$ or schoolchild* or toddler$ or boy or boys or girl* or middle school* or pubescen* or juvenile* or teen* or youth* or high school* or adolesc* or pre-pubesc*).mp. or (child* or adolesc* or pediat* or paediat*).jn.
14     12 and 13
15     12 not 14
16     limit 14 to "all adult (19 plus years)" [Limit not valid in CDSR,ACP Journal Club,DARE,CCTR,CLCMR; records were retained]
17     15 or 16
18     remove duplicates from 17
